# Anxiety, Depression and Quality of Life in Pulmonary Hypertension: A Comparison of Incident and Prevalent Cases

**DOI:** 10.1159/000524369

**Published:** 2022-04-27

**Authors:** Elena Pfeuffer-Jovic, Franziska Joa, Michael Halank, Jens-Holger Krannich, Matthias Held

**Affiliations:** ^a^Department of Internal Medicine, Respiratory Medicine and Ventilatory Support, Medical Mission Hospital, Central Clinic Würzburg, Academic Teaching Hospital of the Julius Maximilian University of Würzburg, Würzburg, Germany; ^b^Department of Pneumology, Internal Medicine I, University Hospital Carl Gustav Carus, Technical University Dresden, Dresden, Germany; ^c^Department of Human Resource Development, Hospital of Julius Maximilian University of Würzburg, Würzburg, Germany

**Keywords:** Depression, Anxiety, Quality of life, Pulmonary hypertension, Incidence, Prevalence, Mental health

## Abstract

**Introduction:**

Anxiety and depression are common in pulmonary hypertension (PH) and health-related quality of life (HRQoL) is reduced. Sufficient analyses in incident and prevalent patients are lacking, so we provide a comparative analysis of these groups with focus on anxiety, depression and HRQoL.

**Methods:**

Depression, anxiety and HRQoL were retrospectively analyzed by Hospital Anxiety and Depression Scale (HADS) and Short Form 36 questionnaire in 91 prevalent and 21 incident PH outpatients from a German tertiary care center specialized in PH. The acquired data as well as hemodynamic and functional parameters of prevalent and incident cases were compared.

**Results:**

HRQoL was reduced in both cohorts of patients. Incident patients had significantly worse HRQoL in physical dominated scales than prevalent patients (physical component summary score: *p* = 0.02; physical role performance: *p* < 0.01). Depression and anxiety were more pronounced in prevalent patients (elevated depression scales: 28.6% of incident group, 35.2% of prevalent group, elevated anxiety scores: 28.6% of incident group, 39.6% of prevalent group). The groups did not differ in hemodynamic data, but incident patients had significantly lower cardiac biomarkers such as NT-proBNP (*p* = 0.016) and hs-troponin (*p* = 0.017). The time since diagnosis was a predictor of the subscale physical role performance (*p* < 0.001).

**Conclusion:**

Physical domains of HRQoL seem to be more limited in incident patients with PH. Anxiety and depression are frequent in both groups. A screening for anxiety and depression is important from the onset of the diagnosis, and patients should receive appropriate therapy to improve HRQoL, anxiety and depression.

## Introduction

High prevalences of anxiety and depression for pulmonary hypertension (PH) are reported and both are strong predictors of health-related quality of life (HRQoL) in patients with PH [1, 2, 3, 4, 5]. Moreover, anxiety, depression and HRQoL have attributed a prognostic value in PH [6, 7, 8]. Patients with PH often suffer from dyspnea, fatigue and chest pain when the final diagnosis is set [9, 10]. Maybe due to these unspecific symptoms, there is often a delay in diagnosis. The time from beginning of symptoms to diagnosis of PH ranges on average from 18 months in chronic thromboembolic PH (CTEPH) [11] up to 47 months in idiopathic pulmonary arterial hypertension [10]. The delay in diagnosis leads to worsening of cardiac and pulmonary function reflected in an increase of symptoms and higher WHO functional classes [10]. It is unclear, if the delay in diagnosis could also increase the risk of developing anxiety and depression. On the contrary, mental disease and reduced HRQoL could accumulate throughout the course of disease, which would lead to a higher frequency of anxiety, depression and diminished HRQoL in prevalent patients.

To our knowledge, there is only one published analysis that deals with the comparison of anxiety and depression in incident and prevalent patients with PH. Somaini et al. [8] reported that incident PH patients suffered significantly more often from anxiety and depression than prevalent PH patients. As far as we know, other studies dealing with the questions of mental disorders and HRQoL in incident and prevalent PH cohorts are lacking.

We already analyzed the groups of PAH and CTEPH of the present cohort in an earlier published study and could show that anxiety and depression are very common in these subpopulations and that both groups suffer from poor HRQoL [12]. In the current study, we compared incident and prevalent patients with PH with the purpose of analyzing HRQoL, anxiety and depression in these groups.

## Materials and Methods

Patients diagnosed with PH, including all groups of PH, were recruited when they had been initially diagnosed or presented for follow-up assessment between August 2010 and December 2011 at the Medical Mission Hospital, a tertiary PH center in Germany. A part of this cohort was partially analyzed before, regarding anxiety, depression and HRQoL in CTEPH and PAH patients in an earlier published study [12].

Recruited patients were to be at least 18 years old. For proper diagnostic approach and treatment, the assessment and therapy were conducted in accordance with current guidelines [13]. Diagnosis of PH was either confirmed or established for the first time by right heart catheterization as recommended [13, 14]. Patients with newly diagnosed PH according to right heart catheterization after first assessment were categorized as incident patients. Patients with the diagnosis of PH for at least 3 months were defined as prevalent patients.

Echocardiography (Vivid7®, GE) and right heart catheterization were performed as recommended [13, 14, 15]. In addition, diagnosis of CTEPH included ventilation-perfusion scan, computed tomography angiography and conventional pulmonary angiography [13].

During follow-up or first assessment, anthropometric data, hemodynamic data and other recommended variables were obtained [13]. The current WHO functional class was assessed in prevalent and incident patients. For prevalent patients, the initial WHO functional class at the time of diagnosis was also recorded. The 6-min walking test (6MWT) was performed as suggested by ATS recommendations [16]. The assessment of dyspnea and exertion was made using the Borg Dyspnea and Exertion Scale, which ranges from 0 to 10 [16, 17, 18, 19]. To complete measurement of functional capacity, cardiopulmonary exercise testing was performed including peak oxygen consumption [19, 20].

High-sensitive troponin (hs-Tn), uric acid and N-terminal prohormone of brain natriuretic peptide (NT-proBNP) were detected using ECLIA (Elecsys 2010, Roche Diagnostics Mannheim, Germany). HRQoL was assessed by using the Short Form 36 (SF-36) questionnaire [21]. This tool consists of 36 items dealing with HRQoL. The 36 items are transformed to 8 subscales and 2 sum scales scoring from 0 to 100. A lower score represents a worse quality of life. Predefined criteria by manual guide were used to analyze the questionnaire [21].

The Hospital Anxiety and Depression Scale (HADS-D, German version) [22] was used to determine anxiety and depression. HADS-D contains in total 14 items, of which respectively 7 are used to represent the anxiety or depression scale. Each scale ranges from 0 to 21, and a cut-off score in HADS-D of 8 or more was used to define patients with elevated anxiety or depression scales [22].

In the prevalent population as well as in the incident population, right heart catheterization and other functional measures for disease severity were performed contemporaneously to HRQoL and HADS-D questionnaires. Survival was documented during the following regular visits at our specialized center and the probability of survival was analyzed according to Kaplan-Meier method.

Statistical analysis was performed using SPSS (Version 20; SPSS Inc., Chicago, IL, USA). Descriptive statistics were used for demographics, and HRQoL was expressed as mean ± standard deviation. Differences in mean scores between incident and prevalent patients were compared through unpaired *t* tests.

Fisher's exact test was used to analyze association in categorical data and Mann-Whitney U test was used to compare ordinal variables. The paired-sample sign test was used to determine a difference in WHO functional class in prevalent patients over time course. A stepwise multiple regression analysis was used to investigate if the time since diagnosis is a predictor of HRQoL.

Written informed consent was obtained from all patients. This study was approved by the local Ethics Committee of the Julius Maximilian University of Wuerzburg (Ethic Committee Number 253/12) and was performed according to the Declaration of Helsinki.

## Results

The cohort consisted of 91 (81.3%) prevalent and 21 (18.7%) incident patients. Eighty-three percent of the whole collective suffered from either PAH or CTEPH. The remaining patients were part of other forms of PH (PH due to lung diseases and/or hypoxia, PH with unclear and/or multifactorial mechanisms). The prevalent patients had been diagnosed for 19 months on average before analysis (median 13 months, range 156 months). The prevalent cohort consisted mainly of 64% of patients with PAH and 22% of patients with CTEPH. In the incident cohort, 57% of the patients were diagnosed PAH and 14% suffered from CTEPH. In the prevalent group, 70% (*n* = 64) were treated for PH with specific medication. Most patients received phosphodiesterase-5 inhibitors (68.8%) and endothelin-receptor-agonists (42.2%). There were 2 patients on calcium channel blocker therapy and 1 patient received inhaled iloprost next to an endothelin-receptor-agonist and phosphodiesterase-5 inhibitors. Two patients with CTEPH had undergone pulmonary endarterectomy with postoperative recurrent PH.

In the incident group, 3 patients (15.8%) were classified as WHO functional class II, whereas 16 patients (84.2%) belonged to WHO functional class III. In the prevalent group, 33 patients (37.1%) were defined as WHO functional class II and 56 patients (62.9%) were assigned to WHO functional class III. The distribution of WHO functional classes between incident and prevalent patients did not reach statistical significance (*p* = 0.28). Regarding the initial WHO functional class of the prevalent cohort at the time of the diagnosis, 12.6% were in WHO functional class II, 82.8% were in WHO functional class III and 4.6% were in WHO functional class IV. The WHO functional classes in the prevalent group were significantly different over time course measured by paired-samples sign test (*z* = −4.07; *p* < 0.001, *n* = 86).

The follow-up investigation revealed that survival probability in the prevalent group was 91.4% after 12 months and 83.2% after 18 months. In the incident group, survival probability was 95.0% after both 12- and 18-month follow-up terms.

Anthropometric, hemodynamic and functional data are shown in Table 1. Sixty-three patients (69.2%) of the prevalent group and 14 patients (66.7%) of the incident group were female. Anthropometric data as well as functional data were not significantly different. Moreover, hemodynamic and echocardiographic parameters such as right atrial area and tricuspid annular plane systolic excursion (TAPSE) did not vary significantly across both cohorts. The cardiac biomarkers NT-proBNP (*p* = 0.02) and hs-Tn (*p* = 0.02) as well as thyroid-stimulating hormone (TSH) (*p* = 0.03) were significantly higher in the prevalent group.

Regarding mental disorders in both groups, 39.6% of the prevalent and 28.6% of the incident patients suffered from elevated anxiety scores in HADS-D. Elevated depression scores were present in 35.2% of prevalent and 28.6% in incident patient group. The comparison of frequency of anxiety and depression − defined by ≥8 points − in the incidence and prevalence group did not reach statistical significance (*p* = 0.46 and *p* = 0.62). Nevertheless, there is a trend toward prevalent patients having more often elevated depression and anxiety scores than incident patients (Table 2). In total, 8 patients in the prevalent group and 1 patient in the incident group had already been treated with psychoactive drugs before study analysis. Among the 36 prevalent patients with elevated anxiety scores, 5 patients (14%) had been under treatment with psychoactive drugs, whereas 2 patients (6%) were treated in the group with elevated depression score (*n* = 32). In the group of incident patients, none of the patients with elevated anxiety and/or depression scores had been treated pharmacologically due to the mental disorder.

HRQoL is reduced in both groups, which is demonstrated in Table 3. The comparison of the subscales between incident and prevalent patients showed that the incident group scored mostly lower than the prevalent group in physical scales. The difference was significant for both “physical role functioning” (*p* < 0.01) and “physical component summary score” (*p* = 0.02).

To evaluate the potential impact of the time of diagnosis on HRQoL, we conducted a stepwise linear regression with 6MWD, NT-proBNP, hs-Tn, age, number of PH-specific drugs and time since diagnosis as possible independent predictors. The stepwise regression analysis could show a correlation between “physical role functioning” and time since diagnosis (in months). Time since diagnosis did not serve as a predictor for the other subscales of HRQoL. Among the other variables, 6MWD was a predictor of HRQoL in almost every scale (Table 4).

## Discussion

Data about anxiety, depression and HRQoL in incident and prevalent cohorts with PH are rare. In our cohort, hemodynamic data did not differ across the groups. Laboratory parameters such as hs-Tn and NT-proBNP were significantly higher in the prevalent group. Nevertheless, incident patients showed a worse WHO functional class than prevalent patients in our cohort. Previous comparisons of incident and prevalent patients reported that mean pulmonary arterial pressure (PAPm) tends to be higher in the prevalent cohort [23, 24, 25]. In the Czech cohort, prevalent patients with PAH also had a higher pulmonary vascular resistance (PVR) than incident patients [24]. Regarding literature, incident patients have even worse WHO functional class status than prevalent patients [8, 23, 24, 25]. Our data could also show this trend, but the comparison did not reach statistical significance. The fact that prevalent patients tend to be in a better WHO functional class, even when they had worse cardiac biomarkers in comparison with incident patients, can possibly be explained by the fact that there is a selection of patients with better prognosis and long-term survivors in the prevalent group. Regarding the initial WHO functional class of the prevalent patients, WHO functional class did improve over the time course.

Strange et al. [10] showed that during the diagnostic period of more than 3 years from the onset of symptoms to diagnosis in idiopathic pulmonary arterial hypertension, WHO functional classes deteriorated up to the time of diagnosis. During this delay, adequate medical therapy is lacking until diagnosis is made. The widespread use of special PAH medication in the prevalent group could therefore result in better WHO functional class [26, 27].

The survival analysis revealed that the probability of survival of the incident patients was slightly higher than the probability of the prevalent group. But an adequate comparison of survival between these groups is not possible due to several reasons. The prevalent cohort had already been diagnosed for 19 months on average before analysis and therefore suffered with high certainty longer from PH than incident patients, which implies a lower survival rate from the time of the beginning of the follow-up. On the one hand, some patients of those who were diagnosed before the start of the analysis and with a worse prognosis had already died before the analysis was conducted. On the other hand, the incident patients are treatment naïve and therefore the initiation of specific medication for PH should lead to an improvement of outcome. The reported survival probabilities of both cohorts should be interpreted with caution due to the mentioned reasons which are caused by the fact that both groups are at different stages of disease.

In the German population, the prevalence of major depressive disorder during 12 months was reported 27.7% in a nationwide survey published in 2014; nevertheless, the prevalence of unipolar depression was estimated at 7.7% and that of anxiety disorders at 15.3% [28]. It is already known that mental disorders appear more often in chronic diseases than in healthy population [29, 30]. Nevertheless, it is remarkable that anxiety and depression are already traceable in 28.6% of the patients with newly diagnosed PH although a concomitant comorbidity as depression or anxiety has not yet been reported. This leads unavoidably to the conclusion that none of the mental comorbidities were treated in any way up to the time of diagnosis. Although the distribution of mental disorder in incident and prevalent cases could not reach statistical significance, there was a trend toward prevalent patients suffering more often from anxiety and depression than incident patients.

Similar facts were already observed in patients with COPD. Anxiety and depression scores of COPD patients deteriorated yearly during the follow-up of 5 years [31]. Moreover, it was reported that during the first year of diagnosis, the risk of developing depression is the highest [32], which underlines the importance of screening for mental disorders in chronic diseases.

Until now, only one study analyzed anxiety and depression in incident and prevalent patients with PH. Somaini et al. [8] investigated mental disorders in a cohort of patients with PAH and CTEPH. Anxiety and depression were reported in 51% and 53% in incident patients, whereas 24% and 21% of prevalent PH patients suffered from anxiety or depression. The prevalence of mental disorders is reported much higher for incident patients than in our cohort. One reason for this could be a lower cut-off value to define elevated HADS in the mentioned study. In the mentioned study, the authors showed an improvement of HADS scores during 12 months after diagnosis of PH [8]. This observation stands in contrast to the assumption that mental disorders become more frequent during time course of a chronic disease [31]. Nevertheless, the reported follow-up results are limited and as the follow-up cohort declined to only a marginal part of the initially analyzed incident patient group [8].

Regarding HRQoL in the cohort of Somaini et al. [8], incident patients scored significantly worse in all HRQoL subscales. This observation is partly confirmed by results of Roman et al. [33]. In our study, HRQoL is diminished in both prevalent and incident groups. Nevertheless, incident patients with PH scored higher in mental subscales than prevalent patients, which goes in line with the distribution of anxiety and depression in these groups in our study. Physical dimension scales were significantly higher for prevalent patients in our study, although 6MWD was similar in both groups. But prevalent patients tend to be more often in a better WHO functional class. As WHO functional class reflects physical activity and correlates well with physical domains in SF 36 [33, 34], the fact that prevalent patients scored higher in the physical dominated domains is comprehensible. The differences in physical domains of HRQoL may also be attributed to medical therapy of PAH, which is known to improve HRQoL [7, 35], and in particular the physical domains of HRQoL [36]. In our study, the newly diagnosed patients have not been receiving any PAH-specific medication so far. The 6MWD seems to be a strong predictor of HRQoL in patients with PH [37]. In the conducted multiple regression analysis, 6MWD was a predictor of HRQoL in every scale except the mental component summary score. For the mental component summary score, none of the independent variables could serve as a predictor in a significant model.

Hs-Tn is already known to be a predictor of outcome in PAH [38]. In our analysis, hs-Tn served as a predictor in HRQoL next to 6MWD in the subscale social functioning (*p* < 0.001).

The time since diagnosis was also a predictor of HRQoL in the scale “physical role functioning” (*p* < 0.001). 6MWD and time since diagnosis could explain 19% of the variance of physical role functioning. Regarding the comparison of the scale “physical role functioning” in prevalent and incident patients, prevalent patients scored higher than incident patients, which goes in line with the time of diagnosis serving as a predictor in the regression analysis of this subscale.

One limiting factor of the study is the use of a generic HRQoL measurement tool instead of a disease-specific assessment tool, which may reflect clinical status more adequately. Nevertheless, the first disease-specific HRQoL assessment tool at that time was CAMPHOR, which was generated in 2006, but was adapted for German-speaking countries not until 2012 [39, 40]. Therefore, we decided to use SF-36 questionnaire to assess HRQoL in PH as it is a very common HRQoL tool which was used worldwide at that time in patients with PH for evaluating HRQoL as study endpoints.

Another limitation of the study is that different forms of PH were included in this monocentric analysis. Perhaps, some effects could be superimposed by inhomogeneity of groups, as, for example, PH due to left heart disease or PH due to lung disease implies other pathophysiologic mechanisms and symptom burden than PAH and CTEPH [27]. Another important fact implying the heterogeneity of the cohort is that PH-specific treatment is only available for patients with PAH and CTEPH and could therefore have an impact on the hemodynamic, functional and possibly HRQoL results of the prevalence group. Nevertheless, the heterogenic cohort represents the typical pattern of PH patients in our specialized tertiary care center. Moreover, the analysis mainly consists of PAH and CTEPH patients with 83%. But meanwhile, there are more treatment options for patients with PAH and CTEPH since the analyzation contains data from August 2010 and December 2011. It could be possible that due to new therapeutic options, the prevalence of anxiety and depression could be reduced and HRQoL could be improved in treated patients. To gain further information about treatment possibilities and effects in this complex interaction, a longitudinal study with follow-up of the incident patients in a homogenic cohort of PAH or CTEPH patients could help.

## Conclusion

This study shows that anxiety and depression can often be found in newly diagnosed patients with PH, which emphasizes the importance of screening for mental disease from the onset of diagnosis. We could also show that incident patients had worse scores on physical subscales of HRQoL than prevalent patients, which is in accordance with the distribution of higher WHO functional classes in the incident group. Moreover, the time of diagnosis is a predictor of HRQoL next to 6MWD and hs-Tn. These results suggest that a rapid diagnosis of PH and screening for mental disease with provision of PH-specific treatment and psychological intervention if needed may help improve anxiety, depression and quality of life in patients with PH.

## Statement of Ethics

All procedures performed in studies involving human participants were in accordance with the ethical standards of the institutional and/or national research committee and with the 1964 Helsinki Declaration and its later amendments or comparable ethical standards. This study was approved by the local Ethics Committee of the Julius Maximilian University of Wuerzburg (Ethic Committee Number 253/12). Consent to participate statement: informed consent was obtained from all individual participants included in the study.

## Conflict of Interest Statement

Dr. Pfeuffer-Jovic reports fees for travel/accommodation from Actelion, Boehringer Ingelheim, Novartis and OMT, outside the submitted work. Dr. Joa has nothing to disclose. Dr. Krannich has nothing to disclose. PD. Dr. Halank reports personal fees for lectures and consultations and travel/accommodation, meeting expenses from Acceleron, Actelion, AstraZeneca, Bayer, Berlin-Chemie, GSK, MSD, Novartis and OMT, outside the submitted work. PD. Dr. Held reports grants from Actelion; honoraria for lectures from Actelion, Bayer HealthCare, Berlin-Chemie, Boehringer Ingelheim, GSK, Novartis and Pfizer; honoraria for advisory board activities from Actelion, Bayer HealthCare, GSK and MSD; and participation in clinical trials of Actelion, Bayer HealthCare, GSK, Pfizer and United Therapeutics, outside the submitted work.

## Funding Sources

There was no funding of the presented work.

## Author Contributions

Writing and original draft: Elena Pfeuffer-Jovic and Matthias Held. Writing and review and editing: Elena Pfeuffer-Jovic, Matthias Held, Franziska Joa, Michael Halank and Jens-Holger Krannich. All authors were actively contributing to interpretation of the results and discussion of the findings. They all approved the final version of the manuscript.

## Data Availability Statement

All data analyzed during this study are included in this article. Further inquiries can be directed to the corresponding author.

## Figures and Tables

**Table 1:** Anthropometric, hemodynamic parameters, functional capacity and cardiac biomarkers of incident and prevalent patients

	Incident patients		Prevalent patients		*p* value
	*N*	mean ± SD	*N*	mean ± SD	
Anthropometric data					
Age, years	21	67.43±9.421	91	69.93±10,975	0.34
BMI, kg/m^2^	21	27.86±5.46	80	28.13±6.35	0.86
Right heart catheterization					
PAPm, mm Hg	21	36.14±10.03	91	38.01±11.35	0.49
PVR, dyn/cm^5^/s	20	412.45±142.61	85	477.69±345.76	0.41
Cardiac index, L/min/m^2^	20	2.75±0.79	84	2.73±0.73	0.90
RAP, mm Hg	21	10.38±5.03	81	11.96±5.16	0.21
PAWP, mm Hg	19	11.32±5.40	87	12.16±5.54	0.48
Echocardiography					
RA area, cm^2^	21	21.62±7.53	90	22.02±8.79	0.85
TAPSE, cm	21	2.24±0.70	90	2.40±1.57	0.65
6MWT					
6MWT, m	21	387.86±90.56	90	370.51±125.70	0.5
Cardiopulmonary exercise test					
VO2-peak, %	16	71.63±15.72	69	73.29±23.95	0.792
VO2-peak, mL/min/kg	17	13.53±2.76	69	13.93±4.31	0.72
Laboratory parameters					
NT-proBNP, pg/mL	21	968.48±784.79	88	1,805.51±2,758.15	**0.02**
hs-Tn, pg/mL	21	12.29±10.69	88	19.91±18.91	**0.02**
TSH, mIU/L	20	1.70±0.73	47	2.62±2.62	**0.03**
Borg scales					
Borg dyspnea score	18	3.50±1.89	85	3.47±2.42	0.96
Borg exertion score	18	3.61±1.82	85	2.54±2.49	0.09

Values are mean, standard deviation and *p* values. Significant values are given in bold. BMI, body mass index; PAPm, mean pulmonary arterial pressure; PVR, pulmonary vascular resistance; RAP, right atrial pressure; PAWP, pulmonary arterial wedge pressure; TAPSE, tricuspid annular plane systolic excursion; VO2-peak, peak oxygen uptake; NT-proBNP, N-terminal prohormone of brain natriuretic peptide; hs-Tn, high-sensitive troponin; TSH, thyroid-stimulating hormone; RA, right atrial.

**Table 2:** HADS score in incident and prevalent patients

		Incident patients	Prevalent patients
*HADS-D anxiety score* HADS score <8	Patients, *n* In incident/prevalent patients, %	15 71.4	55 60.4

HADS score ≥8	Patients, *n* In incident/prevalent patients, %	6 28.6 *p* = 0.46	36 39.6

*HADS-D depression score* HADS score <8	Patients, *n* In incident/prevalent patients, %	15 71.4	59 64.8

HADS score ≥8	Patients, *n* In incident/prevalent patients, %	6 28.6 *p* = 0.62	32 35.2

*p* value calculated by Fisher's exact test.

**Table 3:** SF-36 scales in incident and prevalent patient groups

SF-36 scale	Incident patients		Prevalent patients		*p* value
	*N*	mean±SD	*N*	mean±SD	
Physical functioning	19	39.36±17.78	87	40.62±29.42	0.81
Physical role performance	18	16.67±29.70	80	40.52±44.82	**<0.01**
Bodily pain	20	53.45±29.95	85	65.26±31.19	0.13
General health perception	18	40.54±17.22	84	46.53±19.12	0.22
Vitality	19	43.16±11.69	85	46.27±20.78	0.37
Social functioning	20	73.75±22.54	88	70.88±25.34	0.64
Emotional role performance	19	57.89±46.93	78	58.12±46.97	0.99
Mental health	19	71.16±15.41	84	65.12±18.99	0.20
Physical component summary score	17	29.83±8.55	70	36.07±12.47	**0.02**
Mental component summary score	17	50.86±9.60	70	48.11±11.10	0.35

Values are mean ± standard deviation. Significant values are given in bold.

**Table 4:** Regression analysis of SF-36 scales

Dependent variables	Predictors	B	*p* value predictor	Adjusted *R*^2^	df	*F* value	*p* value (associated with *F* value)
Physical functioning	6MWD	0.14	**<0.001**	0.36	1; 100	56.9	**<0.001**

Physical role performance	6MWD	0.14	**<0.001**	0.19	2; 91	11.8	**<0.001**

	Time since diagnosis	0.33	**0.04**				

Bodily pain	6MWD	0.09	**<0.001**	0.12	1; 99	14.0	**<0.001**

General health perception	6MWD	0.06	**<0.001**	0.14	1; 97	17.2	**<0.001**

Vitality	6MWD	0.06	**<0.001**	0.11	1; 99	13.5	**<0.001**

Social functioning	6MWD hs-Tn	0.42 −0.44	**0.036 0.003**	0.17	2; 101	11.1	**<0.001**

Emotional role performance	6MWD	0.09	**0.02**	0.48	1; 91	5.6	**0.020**

Mental health	6MWD	0.03	**0.036**	0.03	1; 98	4.5	**0.036**

Physical component summary score	6MWD	0.06	**<0.001**	0.34	1; 82	42.8	**<0.001**

Only significant models and predictors are shown. B, coefficient; df, degrees of freedom.
